# Which is the best postoperative chemotherapy regimen in patients with rectal cancer after neoadjuvant therapy?

**DOI:** 10.1186/1471-2407-14-888

**Published:** 2014-11-27

**Authors:** Peng Gao, Yong-xi Song, Jing-xu Sun, Xiao-wan Chen, Ying-ying Xu, Jun-hua Zhao, Xuan-zhang Huang, Hui-mian Xu, Zhen-ning Wang

**Affiliations:** Department of Surgical Oncology and General Surgery, the First Hospital of China Medical University, 155 North Nanjing Street, Heping District, Shenyang, 110001 PR China

**Keywords:** Rectal neoplasms, SEER program, Chemotherapy, Neoadjuvant therapy

## Abstract

**Background:**

There is no general agreement about whether patients who have already received neoadjuvant chemoradiotherapy need further postoperative chemotherapy based on 5-fluorouracil(5-FU) or 5-FU plus oxaliplatin.

**Methods:**

Medicare beneficiaries from 1992 to 2008 with Union for International Cancer Control ypStages I to III primary carcinoma of the rectum who underwent 5-FU-based neoadjuvant chemoradiotherapy and surgery for curative intent were identified through the Surveillance, Epidemiology, and End Results (SEER)-Medicare-linked database. A Cox proportional hazards model and propensity score-matched techniques were used to evaluate the effect of treatment on survival.

**Results:**

For patients with resected rectal cancer who have already received 5-FU-based neoadjuvant chemoradiotherapy, postoperative 5-FU-based chemotherapy did not prolong cancer-specific survival (CSS) in ypStage I (*P* = 0.960) and ypStage II (*P* = 0.134); however, it significantly improved the CSS in ypStage III (hazard ratio = 1.547, 95% CI = 1.101-2.173, *P* = 0.012). No significant differences in survival between the 5-FU group and oxaliplatin group were observed.

**Conclusions:**

For patients with resected rectal cancer who have already received 5-FU-based neoadjuvant chemoradiotherapy, postoperative 5-FU-based chemotherapy prolongs the CSS of groups in ypStage III. Adding oxaliplatin to fluoropyrimidines in the postoperative chemotherapy did not improve the CSS for patients who received neoadjuvant chemoradiotherapy.

**Electronic supplementary material:**

The online version of this article (doi:10.1186/1471-2407-14-888) contains supplementary material, which is available to authorized users.

## Background

Rectal cancer has been defined as a cancerous lesion located within 12 cm of the anal verge [1]. At present, the main treatment for locally advanced rectal cancer is chemoradiotherapy plus total mesorectal excision (TME). Although it is debatable whether preoperative chemoradiotherapy improves long-term survival [2–4], randomized clinical trials have shown better local control, lower toxicity, and higher compliance if preoperative chemoradiotherapy is administered rather than postoperative conventionally fractionated chemoradiotherapy [5, 6]. Thus, the current “gold standard” of treatment recommended by both the National Comprehensive Cancer Network (NCCN) [7] and the European Society for Medical Oncology (ESMO) [8] for locally advanced rectal cancers with invading through the muscularis propria into the pericolorectal tissues (cT3), penetrating to the surface of the visceral peritoneum (cT4a), invading or being adherent to other organs or structures (cT4b), or lymph nodal metastasis on imaging (cN1-2) is preoperative radiotherapy plus 5-fluorouracil (5-FU)-based chemotherapy.

Is adjuvant chemotherapy needed after curative surgery for rectal cancer patients who have received neoadjuvant chemoradiotherapy? The NCCN recommended postoperative chemotherapy for all patients undergoing preoperative chemoradiotherapy regardless of the pathological stage [7]. The ESMO guidelines state that “similar to the situation in colon cancer Stages III (and “high-risk” Stage II), adjuvant chemotherapy can be provided, even if the scientific support for sufficient effect is less” [8]. However, several studies questioned the use of adjuvant chemotherapy in patients with rectal cancer who underwent neoadjuvant chemoradiotherapy and curative surgery, especially in patients without pathological lymph node metastasis (ypN0) [2, 9–14]. Results from all these studies showed that adding postoperative adjuvant chemotherapy did not significantly improve disease-free survival (DFS) or overall survival (OS) in patients who have already received neoadjuvant chemoradiotherapy. On the other hand, a unique randomized clinical trial suggested that good-prognosis patients (ypT0-2) benefit from postoperative chemotherapy [15]. Furthermore, it was not reported whether adding postoperative 5-FU-based chemotherapy could improve the survival of patients with pathological lymph node metastasis (ypN1-2). Hence, for patients who have received neoadjuvant therapy, the role of postoperative chemotherapy is still controversial.

Although lack of data from rigorous randomized clinical trials confirmed the effectiveness, oxaliplatin has been used in rectal cancer for several years based on the extrapolated data in colon cancer. Similarly, the role of postoperative oxaliplatin in patients with rectal cancer who have already received neoadjuvant chemoradiotherapy is still not yet defined. To the best of our knowledge, only two ongoing randomized clinical trials presented preliminary results, though contradictory, on this issue [16, 17].

The aim of the current study was to investigate whether postoperative 5-FU-based chemotherapy or 5-FU plus oxaliplatin provides a benefit for patients with resected rectal cancer who have already received 5-FU-based neoadjuvant chemoradiotherapy.

## Methods

### Data source

The data from the Surveillance, Epidemiology, and End Results (SEER)–Medicare-linked database were examined. The approval and subsequent access to the data for this study were granted by the National Cancer Institute (NCI) and Information Management Services (IMS), Inc. following submission of a formal data request outlining the research objectives. Also, this study was approved by the Institutional Review Board of the first hospital of China Medical University.

The SEER cancer registries include information on patient demographics, tumor characteristics, first course of treatment, and survival of patients who were newly diagnosed with cancer. SEER regions included approximately 26% of the US population [18]. Medicare is the primary health insurer for 97% of the US population aged ≥65 years [19]. The details of the database were presented elsewhere [20].

### Patient selection

All Medicare-enrolled patients aged ≥66 years that were diagnosed with primary adenocarcinoma of the rectum from 1992 to 2008 were included in the study (SEER cancer site codes: 19.9 and 20.9; SEER histology codes: 8000–8152, 8154–8231, 8243–8245, 8250–8576, 8940–8950, and 8980–8981). Those who underwent primary tumor resection with likely curative intent within 180 days of diagnosis were selected, excluding presumably palliative operations. Data of all patients who received preoperative (from diagnosis of rectal cancer to operation) radiotherapy plus 5-FU or capecitabine, which was the regimen recommended by the NCCN, were included. Regarding the postoperative chemotherapy, the no-chemo group included patients with no record for chemotherapy within 120 days of surgery. The oxaliplatin group included patients with any record of oxaliplatin within 30 days of their first chemotherapy dose. The 5-FU group comprised all other patients, including those who received 5-FU or capecitabine. The Health Care Financing Administration Common Procedure Coding System or National Drug Code for drugs were presented in Additional file 1.

Patients were excluded from this study if they (1) received other chemotherapy regimen preoperatively or postoperatively; (2) had prior non-rectal cancer; (3) had incomplete pathological stage entries or diagnostic data; (4) died during the immediate postoperative period (within 30 days); (5) were diagnosed with another malignancy 1 year after the date of rectal cancer diagnosis; (6) had membership in a Medicare-sponsored health maintenance organization or lack of enrollment in Medicare Parts A and B from 12 months preceding diagnosis through 9 months after diagnosis; (7) had complete pathologic response because it was unable to identify accurately in the SEER-Medicare database; and (8) had ypStage 0 (Tis N0 M0), because of too small sample size.

### Variables

Age at diagnosis, year of diagnosis, sex, race, marital status, rural/urban county of residence, census tract-level median household income, and level of education (percentage of people aged >25 years and <12 years of education) were obtained from the SEER patient entitlement and diagnosis summary file. For risk adjustment, Centers for Medicare and Medicaid Services Hierarchical Condition Categories (HCC) based on outpatient and inpatient diagnoses from the 12 months before rectal cancer diagnosis were used. The resulting score can be interpreted as a patient’s predicted level of “future health care need” relative to the average Medicare beneficiary (HCC = 1.0) [21].

Patients were staged according to the seventh edition of the Union for International Cancer Control (UICC) tumor-node-metastasis (TNM) staging system [22]. Postoperative pathological stage (ypTNM) was used. The preoperative clinical stage was not available in the SEER-Medicare. Other covariates included were tumor grade, histological type, preoperative intestinal obstruction, preoperative intestinal perforation, postoperative radiotherapy, and the number of lymph nodes examined.

### Statistical analysis

In the univariable analysis, the cancer-specific (CSS) was analyzed by Kaplan-Meier survival curves, and comparisons were made by the log-rank test stratified by the ypTNM stage.

In clinical practice, significant differences exist between patients who are and are not treated with chemotherapy, particularly with regard to age and comorbidities. Because treatment effect estimates are likely confounded by factors related to treatment selection, a propensity score (PS)-matched analysis was performed to compare the effect of treatment on survival among patients of similar risk profiles as assessed by measured known confounders [23, 24]. For this analysis, logistic regression models were built for each stage to estimate each patient’s probability of receiving 5-FU or oxaliplatin, conditional on covariates. Later two PSs were generated: one estimated the likelihood of 5-FU receipt and the other estimated the likelihood of oxaliplatin receipt in chemotherapy-treated patients. For each comparison, patients exposed to treatment (5-FU and oxaliplatin) were matched with patients with the same PS from the unexposed treatment group. Patients for whom there was no match were excluded. In this manner, a PS-matched cohort balanced across treatment groups for measured confounders was generated. The CSS was then compared in these PS-matched cohorts using the log-rank test stratified by the ypTNM stage.

As the sample size was moderate, a Cox proportional hazards model was also used in the adjusted analysis. The covariates included all variables that were identified to be significantly related to survival in the univariable analysis and the tests were made stratified by the ypTNM stage.

All statistical analyses and graphics were performed by the lead author using SAS 9.3 (SAS Institute, Cary, NC, USA), STATA 12.0 software (STATA, College Station, TX, USA), and PASW Statistics 18.0 software (SPSS, Inc., Somers, NY, USA). For all analyses, *P* < 0.05 was considered to indicate a significant result.

## Results

### Patient demographics

A total of 1535 patients with resected rectal cancer who received neoadjuvant chemoradiotherapy regimen recommended by the NCCN were included (Table 1). Details of race, marital status, median household income, level of education, histologic type, and intestinal perforation were not presented in Table 1, because the number of patients in some subgroups was too small and the SEER-Medicare rules require that cell sizes less than eleven in a table must be suppressed.

**Table 1:** **Clinicopathologic features of patients with different chemotherapy regimens**

	No-chemo	5-FU	Oxaliplatin
Gender			
Male	448	321	131
Female	324	219	92
Age at diagnosis, years			
66-70	201	184	105
71-75	234	175	70
>75	337	181	48
Residence location			
Big Metro	383	261	127
Metro or Urban	303	199	77
Less Urban or Rural	86	80	19
Year of diagnosis			
1992-1996	17	10	0
1997-2000	132	115	0
2001-2004	296	251	26
2005-2008	327	164	197
Histologic grade			
Well	59	35	14
Moderate	519	360	146
Poor/Undifferentiated	116	87	37
Unknown	78	58	26
ypT category			
ypT1-2	261	146	50
ypT3	446	367	160
ypT4	65	27	13
ypN category			
ypN0	609	339	134
ypN1a	79	85	34
ypN1b	42	59	26
ypN2a	20	36	16
ypN2b	22	21	13
ypTNM stage			
ypTNM I	233	113	36
ypTNM II	376	226	98
ypTNM III	163	201	89
Intestinal obstruction			
No	677	484	201
Yes	95	56	22
HCC risk score			
1st quartile	210	143	56
2nd quartile	157	131	45
3rd quartile	189	152	67
4th quartile	216	114	55
Number of examined lymph node			
≥12	265	200	106
<12	507	340	117
Postoperative radiotherapy			
No	676	455	208
Yes	96	85	15

*Abbreviation*s: *HCC* Hierarchical Condition Categories, *No-chemo* without postoperative chemotherapy, *5-FU* 5-fluorouracil.

### CSS without chemotherapy or with 5-FU

**Figure 1:**
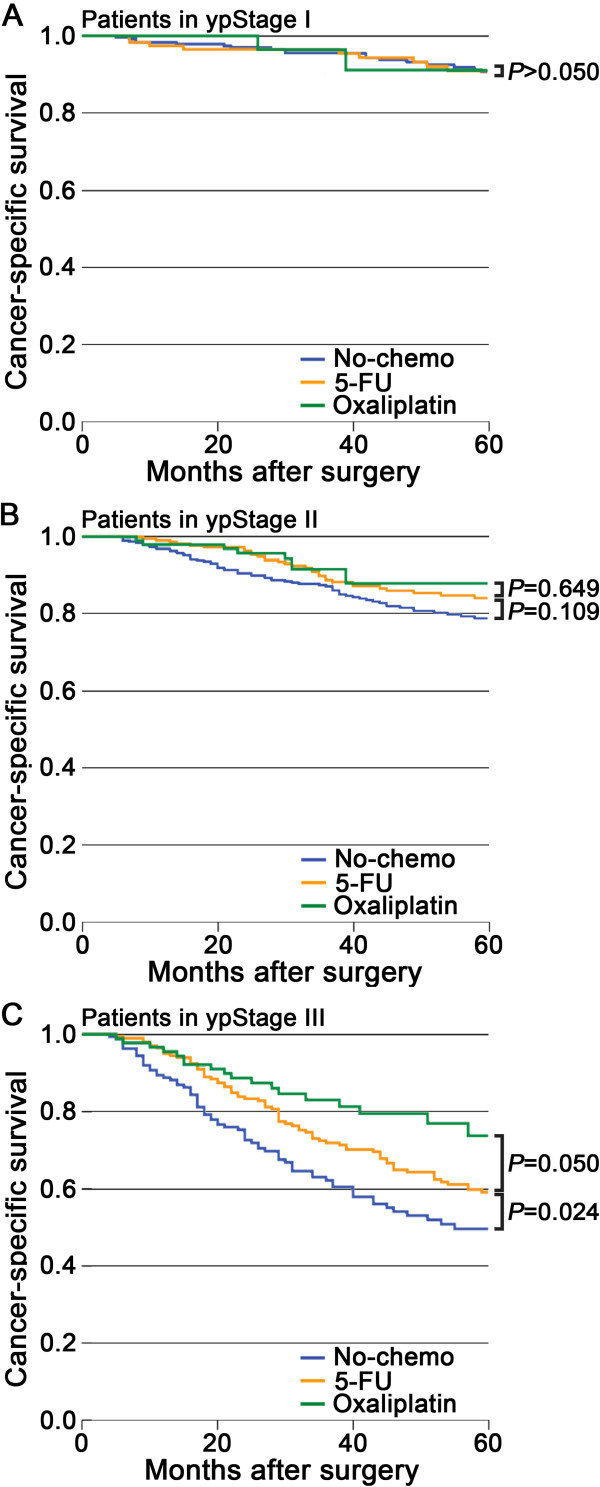
**Kaplan-Meier comparison of cancer-specific survival among patients who received different postoperative treatment stratified by pathologic stage. A.** ypStage I; **B.** ypStage II; **C.** ypStage III.

The unmatched 5-year CSS rates for the patients in the no-chemo group were 90.6%, 78.8%, and 49.5% as compared with 90.9%, 84.0%, and 59.0% for the 5-FU group in ypStage I (T1-2 N0 M0), II (T3-4 N0 M0), and III (Any T N1-2 M0), respectively (Figure 1). There was no significant difference in survival between two groups in ypStage I (*P* = 0.961) and ypStage II (*P* = 0.109). The prognosis of patients in the no-chemo group was significantly worse than the 5-FU group in ypStage III (*P* = 0.024).

**Figure 2:**
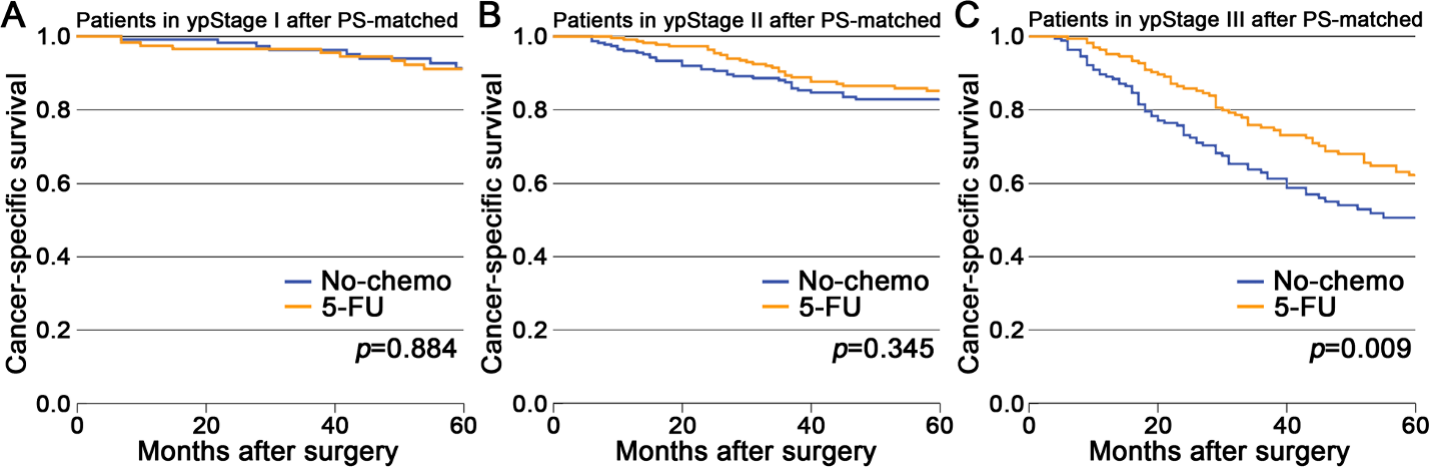
**After PS-matched, Kaplan-Meier comparison of cancer-specific survival between patients in the no-chemo group and in the 5-FU group stratified by pathologic stage. A.** ypStage I; **B.** ypStage II; **C.** ypStage III.

The variables that were significantly related to the patients’ probability of receiving 5-FU were presented in Additional file 2. The PS-matched cohorts were generated using these variables. The CSS was then compared in these PS-matched cohorts. There were still no significant differences in survival between the two groups in ypStage I (*P* = 0.884) and ypStage II (*P* = 0.345), but for patients in ypStage III the prognosis of the no-chemo group was significantly worse than the 5-FU group (*P* = 0.009; Figure 2).

A Cox proportional hazards model was also used for comparison of survival between the two groups. The covariates included all variables that were identified to be significantly related to survival (Additional file 3). The results were consistent with that of the PS-matched analysis, and in ypStage III, the patients in the no-chemo group was significantly worse than the 5-FU group (hazard ratio = 1.547, 95% CI = 1.101-2.173, *P* = 0.012; Table 2).

**Table 2:** **Cox proportional hazards model stratified by ypTNM stage**

	HR	95%CI	P
Stage I			
Chemotherapy regimens			
5-FU	1		
No-chemo	1.021	0.459-2.273	0.960
Oxaliplatin	1.002	0.215-4.670	0.998
Stage II			
ypT category			
ypT3	1		
ypT4a	1.187	0.291-4.841	0.811
ypT4b	1.878	1.058-3.334	0.031
Postoperative radiotherapy			
No	1		
Yes	1.870	1.159-3.018	0.010
Histologic type			
Adenocarcinoma	1		
Mucinous carcinoma	1.877	1.138-3.094	0.014
Signet-ring cell carcinoma	8.078	2.351-27.748	0.001
Chemotherapy regimens			
5-FU	1		
No-chemo	1.393	0.903-2.148	0.134
Oxaliplatin	0.926	0.438-1.956	0.840
Stage III			
ypT category			
ypT1	1.150	0.491-2.692	0.747
ypT2	0.759	0.436-1.321	0.329
ypT3	1		
ypT4a	0.691	0.244-1.959	0.487
ypT4b	1.592	0.934-2.713	0.088
ypN category			
ypN1a	1		
ypN1b	1.233	0.825-1.844	0.306
ypN2a	1.468	0.905-2.381	0.119
ypN2b	2.098	1.310-3.357	0.002
Residence location			
Big Metro	1		
Metro or Urban	1.147	0.822-1.600	0.419
Less Urban or Rural	0.487	0.257-0.923	0.027
Histologic type			
Adenocarcinoma	1		
Mucinous carcinoma	1.466	0.993-2.163	0.054
Signet-ring cell carcinoma	1.220	0.459-3.239	0.690
Histologic grade			
Well	0.716	0.229-2.239	0.565
Moderate	1		
Poor	1.650	0.753-3.619	0.211
Undifferentiated	2.817	0.891-8.910	0.078
Unknown	1.037	0.496-2.171	0.922
Chemotherapy regimens			
5-FU	1		
No-chemo	1.547	1.101-2.173	0.012
Oxaliplatin	0.626	0.372-1.054	0.078

*Abbreviations*: *No-chemo* without postoperative chemotherapy, *5-FU* 5-fluorouracil, *CI* Confidential intervals.

### CSS with or without oxaliplatin

The unmatched 5-year CSS rates for the patients in the oxaliplatin group were 91.1%, 87.9%, and 73.7% as compared with 90.9%, 84.0%, and 59.0% for the 5-FU group in ypStage I, II, and III, respectively (Figure 1). There were no significant differences in survival between the two groups in all stages (*P* ≥; 0.05).

**Figure 3:**
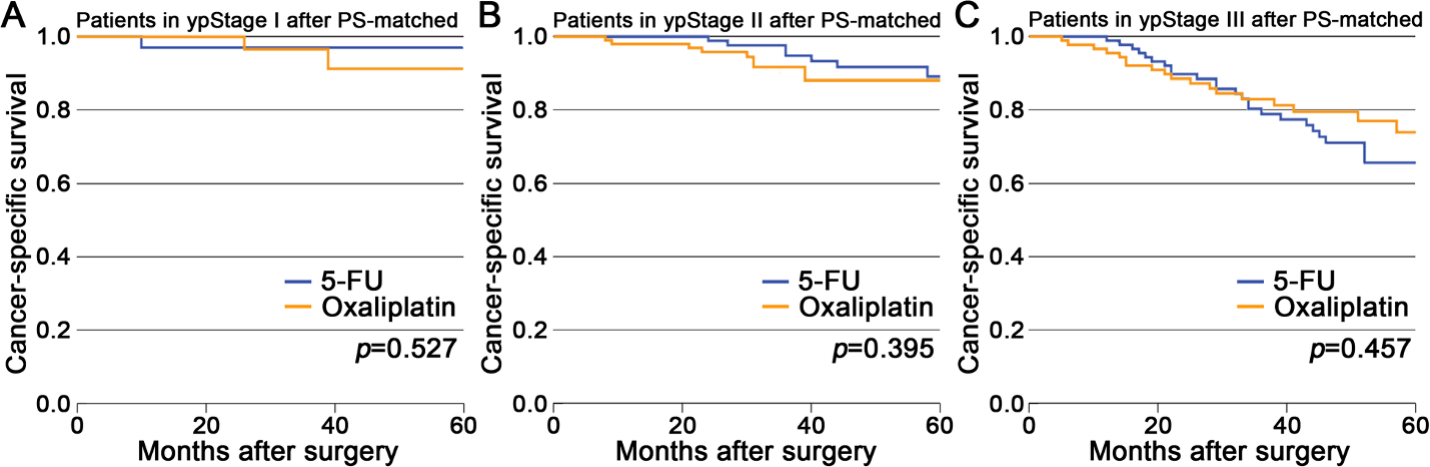
**After PS-matched, Kaplan-Meier comparison of cancer-specific survival between patients in the 5-FU group and in the oxaliplatin group stratified by pathologic stage. A**. ypStage I; **B**. ypStage II; **C**. ypStage III.

The variables that significantly related to the patients’ probability of receiving 5-FU plus oxaliplatin compared with 5-FU alone were presented in Additional file 2. The PS-matched cohorts were generated using these variables. The CSS was then compared in these PS-matched cohorts. There were still no significant differences in survival between the two groups in all stages (*P* > 0.05; Figure 3).

A Cox proportional hazards model was also used for comparison of survival between the two groups. The covariates included all variables which were identified to be significantly related to survival (Additional file 3). The results showed that there were still no significant differences in survival between the two groups in all stages (Table 2).

## Discussion

Preoperative chemoradiotherapy is the standard treatment for locally advanced rectal cancer. However, there is no general agreement about whether patients who have already received neoadjuvant chemoradiotherapy need further postoperative chemotherapy based on 5-FU. Janjan [25] proposed that there was significant improvement in CSS in response to preoperative chemoradiotherapy and the administration of adjuvant chemotherapy. Collette [15] analyzed a subset of data from the European Organization for Research and Treatment of Cancer (EORTC) Trial 22921, which revealed that the postoperative 5-FU-based chemotherapy prolonged survival in ypT0-2, but not in ypT3-4, patients. Hypothetically, distal micrometastasis may be cleared by chemotherapeutic drugs more effectively in patients who were good in response (in low ypStage) to preoperative chemoradiotherapy.

However, Das [26] proposed that postoperative chemotherapy may be of greater benefit for patients in a higher ypStage such as ypStage III (ypN1-2), and lower ypStage subgroups should receive a relatively conservative therapeutic regimen. Subsequently, a study by Huh [11] revealed that postoperative adjuvant chemotherapy for patients in ypT0-2 N0 classification after preoperative chemoradiation and curative surgery did not significantly improve the survival, which was consistent with the suggestion of Das [26], but these results contradictory to that of Collette [15]. Later the results of the study by Govindarajan [9] confirmed that there was no significant difference in the 5-year DFS between patients, in ypT0-2 N0 and ypT3-4 N0 classifications, who did and did not receive adjuvant treatment. In addition, both Fietkau [10] and Kiran [12] proposed that adding postoperative chemotherapy did not significantly improve the survival of patients in ypN0 classification. More recently, Bosset [14] completed the EORTC Trial 22921 and proposed that postoperative 5-FU-based chemotherapy after preoperative radiotherapy (with or without chemotherapy) did not affect DFS or OS. The result was confirmed in both ypT0-2 and ypT3-4 classifications; however, this analysis was not stratified on the basis of ypN classification.

Although the conclusions of the aforementioned studies were different, a consensus may be arrived at based on the postoperative pathologic stage of the patient in determining the need for adjuvant chemotherapy. Simultaneously, several studies proposed that the risk of distant metastases is directly proportional to the postoperative pathologic stage [9, 27–29], and Quah [28] found that the outcome was most accurately estimated by the postoperative pathologic stage. Nevertheless, the NCCN recommended postoperative chemotherapy for all patients undergoing preoperative chemoradiotherapy regardless of the results of the surgical pathology tests [7].

Considering the importance of ypTNM stage in determining the need for adjuvant chemotherapy, all tests were made stratified by ypTNM stage. We compared the prognosis among patients in no-chemo, 5-FU, and oxaliplatin group in the unmatched univariable survival analysis. We found that postoperative 5-FU-based chemotherapy did not prolong the CSS in ypStage I (ypT1-2 N0) (Figure 1A) and ypStage II (ypT3-4 N0) (Figure 1B), which was similar to the study by Govindarajan [9]. On the contrary, adding postoperative 5-FU-based chemotherapy significantly improved survival of patients in ypStage III (ypN1-2) (Figure 1C). To the best of our knowledge, the outcome of postoperative 5-FU-based chemotherapy in ypStage III patients was never reported previously, although researchers stressed the need for a randomized clinical trial [30, 31]. To confirm our results, both the PS-matched analysis and the Cox proportional hazards model were used to make adjusted analysis, and the results were in accordance with the univariable survival analysis (Figure 2, Table 2).

Adding oxaliplatin to fluoropyrimidines in the adjuvant setting improved the OS in colon cancer compared with FU and leucovorin regimens [32]. According to the NCCN guidelines, for patients with resected rectal cancer who have already received 5-FU-based neoadjuvant chemoradiotherapy, 5-FU/leucovorin/oxaliplatin (FOLFOX) was an optional regimen for postoperative chemotherapy. Several ongoing randomized clinical trials in rectal cancer focused on improving 5-FU-based chemotherapy through the addition of oxaliplatin preoperatively, postoperatively, or both [16, 17, 33–35]. Two study groups presented preliminary results. Hong [17] proposed that postoperative FOLFOX significantly improved the 2-year DFS relative to postoperative 5-FU-based chemotherapy for rectal cancer patients in ypStage II or III after 5-FU-based neoadjuvant chemoradiotherapy followed by TME. On the contrary, Nimeiri [16] discovered that there was no difference in the OS between patients who received 5-FU alone or FOLFOX as postoperative chemotherapy. In the current study, no significant differences were found in the survival between the two groups (5-FU vs. oxaliplatin) of patients with resected rectal cancer who have already received 5-FU-based neoadjuvant chemoradiotherapy (Figures 1 and 3; Table 2). Although this result need to be confirmed by further clinical trials, we argue that, for patients who received neoadjuvant chemoradiotherapy, adding oxaliplatin to fluoropyrimidines in the postoperative chemotherapy require serious consideration at present.

The current study has some limitations. First, as it was a retrospective exploratory study, the potential for confounding based on patient selection could not be eliminated. Both traditional Cox proportional hazards model and PS-matched techniques were used to account for known relevant confounders. Second, only patients aged ≥66 years at the time of diagnosis were included in this study, which may limit the applicability of the findings to younger patients with rectal cancer. Third, the role of several known prognostic features such as tumor regression grade, preoperative carcinoembryonic antigen, microsatellite instability, perineural invasion, and lymphovascular invasion could not be investigated, as these characteristics were not available within the SEER-Medicare database. Patients with pCR were excluded from analysis because the pCR status was not well supported by the SEER-medicare database. Fourth, this study retrospectively examined the use of chemotherapy as identified through the Medicare claims data using a “one-claim” algorithm [36, 37]. This created a heterogeneous population in which some patients received a substandard duration of therapy. However, O'Connor [38] proposed that the “none versus any” approach used to assign treatment status provided a window into the effectiveness of chemotherapy in real-world practice, in which an individual’s likelihood of completing the treatment course is not known at the outset of the study. Finally, the preoperative clinical stage as well as pathologic response to neoadjuvant therapy was not supported by the SEER-Medicare database. Both the preoperative clinical stage and pathologic response were related to outcome; however, Quah [28] found that the outcome was most accurately estimated by the postoperative pathologic stage and the clinical stage adds no predictive value to the prognosis. In addition, the 100% accuracy of clinical staging was untenable even in the best of centers [39]. Based on this, the decision of postoperative chemotherapy could be regarded mainly as based on postoperative pathologic stage.

## Conclusions

It is concluded that, for patients with resected rectal cancer who have already received 5-FU-based neoadjuvant chemoradiotherapy, postoperative 5-FU-based chemotherapy prolongs the CSS of groups in ypStage III. Adding oxaliplatin to fluoropyrimidines in the postoperative chemotherapy did not improve the CSS for patients who received neoadjuvant chemoradiotherapy.

### Consent

The manuscript was approved by SEER-Medicare for anonymity prior to submission for publication. Because the SEER-Medicare data are de-identified and are based on registry data, no prior informed consent was required.

## Electronic supplementary material

Additional file 1: Table S1: The Health Care Financing Administration Common Procedure Coding System or National Drug Code for drugs. (PDF 9 KB)

Additional file 2: Table S2: Main effect variables in propensity score models stratified by ypTNM stage. (PDF 148 KB)

Additional file 3: Table S3: Univariate prognostic analysis stratified by ypTNM stage. (PDF 61 KB)

## Abbreviations

TME: Total mesorectal excision
NCCN: National Comprehensive Cancer Network
ESMO: European Society for Medical Oncology
5-FU: 5-fluorouracil
DFS: Disease-free survival
OS: Overall survival
CSS: Cancer-specific survival
SEER: Surveillance, Epidemiology, and End Results
HCC: Hierarchical Condition Categories
UICC: Union for International Cancer Control
TNM: Tumor-node-metastasis
PS: Propensity score
EORTC: European Organization for Research and Treatment of Cancer
FOLFOX: 5-FU/leucovorin/oxaliplatin.
