# Supplementary material for: Which is the best postoperative chemotherapy regimen in patients with rectal cancer after neoadjuvant therapy?
Source: BMC Cancer. 2014 Nov 27;14:888. doi: 10.1186/1471-2407-14-888 (PMC4255436; doi:10.1186/1471-2407-14-888)
Supplement: Supplementary file 1 — Additional file 1: Table S1: The Health Care Financing Administration Common Procedure Coding System or National Drug Code for drugs. (PDF 9 KB) [file 12885_2014_5055_MOESM1_ESM.pdf]

Additional Table 1 The Health Care Financing Administration Common Procedure Coding System or National Drug Code for drugs

Drug	Code
5-FU	J9190, J9200, 00013103691, 63323011710
capecitabine	J8520, J8521, 54868414300, 54868526002, 54868526000, 54868526001, 00004110022, 00004110013, 00004110151, 00004110051, 00004110020, 00004110116, 00004110150
oxaliplatin	J9263, C9205

Abbreviation: 5-FU: 5-fluorouracil;
