# Supplementary material for: Which is the best postoperative chemotherapy regimen in patients with rectal cancer after neoadjuvant therapy?
Source: BMC Cancer. 2014 Nov 27;14:888. doi: 10.1186/1471-2407-14-888 (PMC4255436; doi:10.1186/1471-2407-14-888)
Supplement: Supplementary file 2 — Additional file 2: Table S2: Main effect variables in propensity score models stratified by ypTNM stage. (PDF 148 KB) [file 12885_2014_5055_MOESM2_ESM.pdf]

Additional Table 2 Main effect variables in propensity score models stratified by ypTNM stage

	ypStage I	ypStage II	ypStage III
Variables that significantly related to the patients' probability of receiving 5-FU compared with No-chemo	age at diagnosis, HCC score	pT category, age at diagnosis, sex, years of diagnosis, residence location	age at diagnosis, intestinal obstruction, years of diagnosis
Variables that significantly related to the patients' probability of receiving 5-FU plus oxaliplatin compared with 5-FU alone	years of diagnosis, number of lymph nodes examined	age at diagnosis, years of diagnosis, number of lymph nodes examined, median household income, intestinal obstruction, postoperative radiotherapy, residence location	age at diagnosis, years of diagnosis

Abbreviation: No-chemo, without postoperative chemotherapy; 5-FU: 5-fluorouracil; HCC, Hierarchical Condition Categories.
