# Supplementary material for: Which is the best postoperative chemotherapy regimen in patients with rectal cancer after neoadjuvant therapy?
Source: BMC Cancer. 2014 Nov 27;14:888. doi: 10.1186/1471-2407-14-888 (PMC4255436; doi:10.1186/1471-2407-14-888)
Supplement: Supplementary file 3 — Additional file 3: Table S3: Univariate prognostic analysis stratified by ypTNM stage. (PDF 61 KB) [file 12885_2014_5055_MOESM3_ESM.pdf]

Additional Table 3 Univariate prognostic analysis stratified by ypTNM stage

	ypStage I		ypStage II		ypStage III	
	5-YSR(%)	*P*	5-YSR(%)	*P*	5-YSR(%)	*P*
Gender		0.966		0.905		0.733
Male	90		82		57	
Female	91		81		59	
Age at diagnosis, years		0.096		0.864		0. 679
66-70	94		82		60	
71-75	91		83		58	
76-80	84		80		58	
>80	94		79		56	
Race		0.355		0.572		0.655
White	91		82		58	
Black	81		81		48	
Asian	88		82		66	
Other	100		73		60	
Residence location		0.703		0.133		0.027
Big Metro	89		84		58	
Metro or Urban	91		78		53	
Less Urban or Rural	94		83		77	
Marital status		0.713		0.227		0.928
Single	86		76		58	
Married	92		83		59	
Widow/divorce	88		78		57	
Other	90		100		49	
Median household income		0.525		0.363		0.129
1st quartile	91		83		65	
2nd quartile	88		78		57	
3rd quartile	95		78		48	
4th quartile	88		85		63	
Unknown	100		100		100	
Less than 12 years education		0.915		0.503		0.320
1st quartile	90		85		64	
2nd quartile	92		80		48	
3rd quartile	92		83		56	
4th quartile	89		78		64	
Unknown	100		100		100	
Year of diagnosis		0.203		0.081		0.069
1992-1996	100		70		50	
1997-2000	97		78		50	
2001-2004	88		80		57	
2005-2008	91		85		63	
Histologic type		0.212		<0.001		0.029

Adenocarcinoma	90		84		61	
Mucinous carcinoma	100		67		50	
Signet-ring cell carcinoma	N/A		0		31	
Histologic grade		0.454		0.539		0.001
Well	97		81		69	
Moderate	90		81		61	
Poor	85		77		44	
Undifferentiated	N/A		100		33	
Unknown	95		87		69	
ypT category		0.689		0.001		0.005
ypT1	91		N/A		64	
ypT2	90		N/A		69	
ypT3	N/A		83		58	
ypT4a	N/A		81		67	
ypT4b	N/A		61		29	
ypN category		N/A		N/A		<0.001
ypN0	91		82		N/A	
ypN1a	N/A		N/A		65	
ypN1b	N/A		N/A		60	
ypN2a	N/A		N/A		57	
ypN2b	N/A		N/A		34	
Intestinal obstruction		0.360		0.115		0.379
No	91		82		59	
Yes	84		76		51	
Intestinal perforation		N/A		0.134		N/A
No	91		82		58	
Yes	N/A		50		N/A	
Postoperative radiotherapy		0.262		0.004		0.559
No	90		84		59	
Yes	95		68		54	
HCC risk score		0.447		0.417		0.344
1st quartile	94		84		59	
2nd quartile	90		83		66	
3rd quartile	91		82		55	
4th quartile	88		77		54	
Number of examined lymph node		0.549		0.100		0.533
≥12	92		85		54	
<12	90		80		60	

Abbreviation: N/A, Not Available; HCC, Hierarchical Condition Categories; 5-YSR, Five years survival rate.
